# Consensus on key evaluation tools for stroke: a Delphi study protocol for the assessment of balance and gait

**DOI:** 10.3389/fneur.2026.1764795

**Published:** 2026-03-17

**Authors:** Chiara Castagnoli, Elisa Gervasoni, Davide Cattaneo, Marco Germanotta, Diego Longo, Massimiliano Gobbo, Joel Pollet, Rosa Pullara, Rebecca Cardini, Irene Giovanna Aprile, Marco Baccini, Francesca Cecchi

**Affiliations:** 1 IRCCS Fondazione Don Carlo Gnocchi, Florence, Italy; 2 IRCCS Fondazione Don Carlo Gnocchi, Milan, Italy; 3 Department of Pathophysiology and Transplantation, University of Milan, Milan, Italy; 4 Department of Experimental and Clinical Medicine, University of Florence, Florence, Italy; 5 Department of Clinical and Experimental Sciences, University of Brescia, Brescia, Italy

**Keywords:** neurological rehabilitation, precision medicine, rehabilitation, stroke, stroke rehabilitation

## Abstract

**Background:**

Stroke is a leading cause of long-term disability, and gait and balance impairments significantly affect post-stroke independence and quality of life. Following rehabilitation, patients can regain walking and balance abilities. These gains may occur through true recovery, characterized by the restoration of pre-stroke motor patterns and underlying neural functions and structures, or through compensation, characterized by the use of alternative movement strategies and neural reorganization. Identifying outcome measures that describe gait and balance recovery and distinguish true recovery from compensation could greatly improve the accuracy of functional diagnosis and help tailor rehabilitation by guiding strategy selection. This protocol outlines a Delphi study to establish expert consensus on the most appropriate assessment tools for gait and balance in people with stroke and on their ability to differentiate between recovery and compensation across the International Classification of Functioning, Disability and Health (ICF) domains. The resulting tool set will support individualized, evidence-based rehabilitation strategies.

**Methods:**

The Delphi study employs a two-phase design comprising an initial expert focus group followed by a multi-round electronic Delphi process. The focus group will identify key constructs and candidate tools to assess either recovery or compensation. In the Delphi rounds, a panel of 30 to 50 international experts in stroke rehabilitation will rate the relevance of tools, including clinical scales, instrumented assessments of motor performance, and neural information, across the ICF domains. Consensus will be defined as ≥75% agreement, and iterative rounds will refine item selection based on panel feedback.

**Expected outcomes:**

The study will produce a consensus-based core set of assessment tools for evaluating post-rehabilitation gait and balance recovery after stroke, capable of distinguishing true recovery from compensation. These tools will facilitate more precise diagnosis, patient stratification, and targeted interventions. Additionally, the study will support the design of future research protocols, including cross-sectional and longitudinal studies of stroke rehabilitation outcomes.

**Discussion:**

This study aims to advance precision rehabilitation after stroke by identifying outcome measures that distinguish true recovery from compensatory strategies. A Delphi methodology will provide a rigorous, consensus-driven basis for selecting clinically meaningful tools, thereby enhancing assessment accuracy and potentially improving rehabilitation outcomes.

## 1 Introduction

Stroke remains a leading cause of mortality and long-term disability globally (1). As population growth and aging continue, combined with reductions in stroke-related mortality, the prevalence of individuals living with stroke-related sequelae is rising (2). This trend results in an increased demand for rehabilitation services. In this context, research is essential to enhance clinical practices and ensure that rehabilitation systems can adequately address the complex needs of stroke survivors (3).

Motor impairment is the most prevalent deficit following a stroke, manifesting as loss or limitation in muscle control or mobility (4). Around two-thirds of survivors initially experience mobility impairments, and over 30% remain unable to walk independently 6 months post-stroke (5).

Gait and balance disorders are among the most common and impactful consequences of stroke (6). These impairments significantly hinder independence in activities of daily living and deteriorate quality of life. Post-stroke muscle weakness and loss of voluntary movement contribute to reduced gait speed (7). Moreover, considerable temporal and spatial asymmetries between limbs—reported in 48%–82% and 44%–62% of stroke survivors, respectively—are associated with compromised postural control during ambulation (8). As a result of impaired balance during walking, 70% of stroke survivors living at home report at least one fall within the first year after stroke (6). Thus, enhancing walking ability and restoring balance and movement are fundamental goals of stroke rehabilitation (9–11).

Precision rehabilitation is a promising approach to significantly reduce long-term disability following neurological events such as stroke. As in all branches of precision medicine (12, 13), it aims to provide the most appropriate type and intensity of therapy at the optimal time to maximize functional recovery in individual patients. The idea is theoretically compelling and is already, to some extent, reflected in contemporary clinical practice, where interventions are necessarily adapted to the specific needs of each patient. However, developing a systematic, rigorous, and evidence-based model to support its implementation remains a significant challenge (14). Therefore, there is an urgent need to establish a comprehensive framework for precision rehabilitation that allows for the structured advancement of knowledge in this area through rigorous scientific research and promotes the use of reproducible, evidence-based methodologies that can be systematically tested, evaluated, and refined over time.

Although research on neurorehabilitation has made progress in recent decades, several challenges remain. One central question in rehabilitation research is whether the behavioral improvement observed is due to the recovery of original motor strategies (i.e., true recovery) or to the development of compensatory mechanisms (i.e., *compensation*) (15). In 2004, based on the International Classification of Functioning Disability and Health (ICF) (16) framework, the ICF Core set criteria for stroke assessment were published (17). Five body structure components were identified, including the brain, cardiovascular system, shoulder region, and upper and lower extremities, but most components were selected in the body functions, activities, and participation domains. Later, in a 2009 paper, Levin et al. strongly argued for the need to identify specific assessment tools to measure changes reflecting either recovery or compensation across the ICF domains, to improve the accuracy of functional diagnosis, and to guide individualized rehabilitation planning towards promoting recovery or addressing functionally more beneficial compensatory strategies (18).

In detail, at the neuronal level, recovery entails restoration or reorganization of brain structures, particularly in peri-lesional and diaschisis regions. In contrast, compensation involves recruiting different brain areas to replace the injured ones. At the body function level, recovery is marked by the reappearance of pre-morbid motor patterns, such as active range of motion, muscle tone, motor synergies, and interjoint coordination. At the same time, compensation involves adaptive or substitutive strategies. At the activity level, recovery is defined by task execution using original movement patterns, whereas compensation reflects the use of alternative segments or strategies, often indistinguishable in standard clinical assessments (18).

More recently, the role of neuroplasticity has been clearly stated. Currently, motor recovery after stroke is considered to be driven by different interactive mechanisms, including recovery of perilesional tissue in the first days/weeks after stroke, resolution or alleviation of diaschisis phenomena, homeostatic and learning-dependent neuroplasticity, which can be assessed by neuroimaging, neurophysiologic or serum biomarkers, and, finally, behavioral compensation strategies. These interrelated mechanisms operate in different, although partially overlapping, time windows after the stroke (19). In fact, the indirect monitoring of neuroplastic phenomena using developed and validated biomarkers has been identified as a key feature for improving understanding of the neurobiology of spontaneous and treatment-induced recovery in humans (20), helping distinguish true recovery from compensatory neural phenomena.

Currently, there is a consensus on a basic set of tools for stroke assessment, including those for gait and balance (21, 22) and work is underway to create targeted selection of tools to distinguish recovery from compensation (23–25) to improve the validity and comparability between stroke recovery and rehabilitation studies as a prerequisite for building high-quality, standardized “big data” sets to aid the progress toward precision medicine in stroke rehabilitation.

Expert consensus is needed to select the most informative assessment tools (26), and it is lacking in the use of kinetic and kinematic measures (metrics) for motor recovery (24). The Delphi method, originally developed by the RAND Corporation in the 1950s (27) to facilitate expert consensus through structured, iterative surveys, is a well-established and appropriate approach to address complex, poorly defined clinical questions (26). It is particularly suited to synthesizing diverse expert opinions when empirical evidence alone is insufficient to establish best practices (28). It is widely used to develop clinical standards, measurement tools, guidelines, and expert recommendations, particularly for concepts without universally accepted definitions, thereby facilitating clinical practice in areas that require clarity (21, 28, 29). In this Delphi study, we build on the conceptual distinction between motor recovery and compensation proposed by Levin et al. (18) while adhering strictly to the ICF framework. Accordingly, neural, kinematic, and physiological processes are classified within the Body Function and Structures, reflecting the ICF definition of health condition as the clinical diagnosis (stroke) rather than neural repair or reorganization.

On this ground, the present study aims to identify, through a Delphi process, the best evidence-based tools to assess gait and balance recovery in persons post-stroke, with particular emphasis on distinguishing true recovery from compensation, drawing on the available literature on the subject (25). Consistent with Levin et al. (18), “true recovery” is defined at the motor level as the restitution of lost motor elements, operationalized at the Body Functions and Structures level as “the reappearance of premorbid movement patterns during task accomplishment”. At the neural level, recovery refers to the “restitution or repair of (neural) structures or functions” that support movement. Similarly, “compensation” is defined at the motor level as “the appearance of alternative movement patterns resulting from the adaptation of remaining motor elements or substitution” (18), and, at the neural level, as neural reorganization, reflected by the recruitment of neural structures or functions not predominantly involved prior to the stroke. By establishing expert consensus, we aim to provide clinicians and researchers with additional evidence to select the most effective tools for assessing gait and balance after stroke, ultimately promoting individualized, precision-based rehabilitation strategies.

## 2 Methods

### 2.1 Study design

Following established methodological recommendations (27), this study will use a two-phase approach, comprising an initial focus group pilot and a Delphi consensus panel. The focus group will engage a small subset of panelists comparable to those who will be invited to participate in the Delphi process and will aim to ensure clarity and consistency in data collection procedures, instructions, and question framing (30) and to establish criteria for identifying national and international experts who will be involved in the second phase. Participants in the focus group will also be included in the second phase. Panelists will be invited to provide open-ended feedback on the study topic before the first structured survey round. The Delphi study will consist of multiple iterative rounds of standardized surveys administered to a selected, anonymized panel of experts to reach consensus on the study’s topics (26).

### 2.2 Expert focus group methodology

The focus group, in line with established qualitative research guidelines (31, 32), should include experienced clinicians, researchers, and bioengineers. We have therefore chosen to include the research team, plus a few other experts proposed by team members and recruited for convenience and availability through their work contacts. The discussion will focus on identifying optimal tools for assessing gait and balance in Stroke and on distinguishing between recovery and compensation of these functions (30).

The survey will address three main levels of assessment. The first level will focus on clinical scales, subdivided into measures within the activity domain of the ICF—specifically targeting gait and balance—and measures of body structure and body function that impact these domains. The second level will focus on instrumental tools and protocols for the objective, quantitative evaluation of movement quality. The third level will explore instrumental approaches to investigate neural mechanisms underpinning true recovery or compensation.

The responses collected will be summarized to stimulate further discussion, allowing participants to delve deeper.

The list of clinical scales and instrumental tools to be included in the survey was generated through a structured process, including a literature review to identify measures commonly used in stroke rehabilitation for gait and balance, consultation of dedicated databases such as Stroke Engine and the Shirley Ryan AbilityLab Measures Database, and contributions from the research team to refine and expand the list with clinically meaningful and widely adopted instruments. The resulting list will be provided to respondents as the basis for the survey. To ensure completeness and to capture emerging or less widely disseminated instruments, respondents will also be allowed to suggest additional tools not included in the initial list.

### 2.3 Delphi methodology

The Delphi process will be conducted using Survalyzer (Survalyzer AG, Zurich, Switzerland), an electronic Delphi (E-Delphi) platform designed to facilitate global participation while preserving participant anonymity (27). The features of this platform, including automated data processing and personalized feedback reports, are designed to streamline the process, improve engagement, and minimize logistical barriers and costs associated with international expert panels (33). User-friendly E-Delphi systems will support questionnaire distribution, real-time data analysis, and participant interaction (28).

### 2.4 Delphi expert recruitment

In accordance with Delphi guidelines, we will recruit 30–50 panelists, oversampling to account for anticipated attrition (28). Clear communication regarding study objectives, methods, and timelines will be provided. The panel will include clinicians (neurologists, physicians, occupational and physical therapists), researchers, bioengineers, and rehabilitation specialists with expertise in gait, balance, and stroke from around the world. Recruitment will prioritize diversity in professional background and geographic representation to ensure a broad spectrum of perspectives. We will include demonstrated expertise in stroke rehabilitation, gait, or balance assessment, defined by meeting at least two of the following criteria: (a) a minimum of 5 years of clinical or research experience in the field; (b) authorship of at least three peer-reviewed publications on a relevant topic; (c) holding a leadership position in a relevant professional society or expert group as suggest in literature (34). Recruitment strategies will involve direct outreach to experts identified by the research team. Panelists will be initially identified by the research team and subsequently suggested by the contacted experts using the snowball method for qualitative research, taking care to ensure international representation. We acknowledge that including the research team and experts recruited for convenience in the focus group may introduce selection bias. To mitigate this, the list of tools generated by the focus group will be presented to the Delphi panel as a starting point, and panelists will be explicitly invited to suggest additional tools in Round 1. This will ensure that the final list is not unduly influenced by the initial focus group. Co-authorship opportunities on resultant publications will be offered as an incentive to minimize dropout rates, and active collaboration in the manuscript review process will be offered to the whole group in accordance with ICMJE guidelines.

### 2.5 Delphi administration

**Figure 1:**
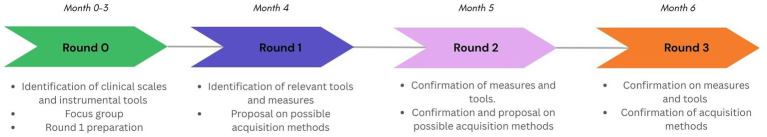
Representation of the Delphi rounds, including a timeline and key points for each round.

Panelists will be sent a list of the tools identified in the focus group, including the results of the literature review. They will be asked to rate each tool on its ability to assess recovery and compensation, and to report any other tools not included in the list if, based on their experience, they deem it appropriate. The Delphi process will include one initial round (Round 0) with the research group to identify the tools to be evaluated. In the first round, panelists will be asked to identify tools that distinguish between recovery and compensation and, if appropriate, propose additional tools not present in the first round. At the beginning of the round, a written introduction to the purpose of the Delphi and its theoretical framework will be provided, and a video-recorded presentation will explain how the Delphi process works, the content of each round, and the requirements for each participant. Subsequently, in the following rounds, rate each measure or tool on a 1–9 point Likert scale for its ability to distinguish recovery from compensation, where 1 = “Not at all able” and 9 = “Extremely able” (Round 2 and 3). The cutoff for including measures or tools in the second round is ≥75% agreement among participants. Also, the measures or tools that receive at least 50% of the choices will also be included in the second round (29). It will be conducted via the E-Delphi platform to enable international participation and streamline data collection. To foster sustained engagement and transparency, panelists will receive feedback reports comparing their responses with the panel’s collective results after each round. Key points and timeline of each round are presented in Figure 1, and in depth as follows:

- Round 0 (Idea generation and initial evaluation): This preparatory phase will combine evidence from the literature and specialized databases with expert input from the research team to identify candidate clinical scales, instrumental movement quality tools, and neural assessment tools. The outcome will be a structured set of items that will serve as the basis for the Round 1 questionnaire.
- Round 1 (assessment): This round includes the collection of panelists’ skills and experience using open-ended questions. The selection of the proposed measures or tools derived from the focus group work using a closed-ended questionnaire (27), the possible addition of more tools, and the suggestion of possible guidelines or references to adopt while using the proposed tools (e.g., “Which of the following instrumented tools do you consider appropriate to distinguish between Recovery and Compensation while assessing BALANCE in individuals post-stroke?”).
- Round 2 (feedback and discussion): Delivery of customized summary reports to the panelists, illustrating their responses in relation to the panel and highlighting the level of consensus, with a subsequent second evaluation based on the responses of the measures and tools identified and the guidelines or references proposed, through a 1–9 point Likert scale. It will be possible to propose other guidelines or references (e.g., “Please indicate your level of agreement that each of the following instrumented tools is appropriate for assessing BALANCE in individuals post-stroke”).
- Round 3 (re-evaluation to reach consensus): A further round is conducted if consensus is not reached, including qualitative feedback to refine items, and a final revision on all measures, tools, guidelines, and references; though a 1–9 point Likert scale.

Panelists will have 1 to 4 weeks to complete each round (1–3).

### 2.6 Consensus definition and Delphi data analysis

The primary outcome is consensus on a core set of measurement tools to assess gait and balance, with the ability to discriminate between recovery and compensation across the ICF domains. The data from each Delphi round will be analyzed independently using descriptive statistics (frequencies and percentages) to assess the level and progression of consensus for each element. Consensus will be defined as ≥75% agreement among panelists, consistent with standard Delphi thresholds (range 50%–97%) (29, 35), and will serve as the *a priori* criterion. We will consider a partial consensus of ≥50% (29). For rounds 2 and 3, consensus will be regarded as if ≥75% of participants rate the measures or tools at 6 or higher on the 1–9 point Likert scale. Agreement will be quantified using measures of central tendency and dispersion (e.g., the median and the interquartile range). In cases of persistent disagreement or polarization (e.g., bimodal distribution of ratings), we will perform pre-planned subgroup analyses to explore potential differences in opinion between professional groups (e.g., clinicians vs. bioengineers). These results will be presented to the panel in subsequent rounds to stimulate discussion and will be reported in the final manuscript. If consensus is unattainable, we will report detailed round-wise results, investigate potential causes for disagreement, and provide a narrative interpretation. Panelist anonymity will be preserved; however, the research team will have access to identities. The research team’s identity will remain undisclosed.

### 2.7 Study timeline

Initial E-Delphi activities began in 2025. Expert recruitment will occur from October to December 2025. Survey rounds will be conducted from January to March 2026. Data analysis is expected to be completed by April 2026.

### 2.8 Ethical considerations

Ethical requirements were assessed according to local regulations, which state that neither formal ethical approval nor informed consent from participants is required for this study, as it is based on expert opinion and does not involve patients or vulnerable individuals. We will consequently inform all panelists that no Ethical requirements are needed. However, we recommend that researchers intending to replicate this study in other jurisdictions consult their local institutional review boards to ensure compliance with local regulations.

### 2.9 Knowledge transfer

The research team engaged in collaborative discussions with clinicians, researchers, engineers, and professionals specializing in gait analysis, balance, and stroke rehabilitation. These interactions helped define the study’s rationale and methodological framework and identify the main theoretical and practical challenges the research sought to address. The survey results will be disseminated through publication in a peer-reviewed journal specializing in rehabilitation research to ensure accessibility to interested parties. Additionally, the results will be presented at national and international scientific meetings. Feedback on the conceptual and methodological definitions developed during the study will be sought from experts in theoretical, clinical, and methodological areas to inform and refine future research directions.

## 3 Discussion

The evaluation of gait and balance in individuals post-stroke needs an evidence-based approach to ensure both accurate assessment and effective rehabilitation planning. Given the complexity and multidimensionality of gait and balance impairments, selecting appropriate assessment tools benefits significantly from expert consensus. Despite the availability of numerous standardized scales and tests, there is no consensus on the most suitable instruments to assess gait and balance in stroke patients, particularly for distinguishing between post-rehabilitation improvements that reflect true recovery and those that reflect compensatory strategies.

The absence of standardized outcome measures within a defined conceptual framework presents significant challenges for comparing study results, tracking patient progress, and designing individualized, targeted rehabilitation interventions. Establishing a shared assessment framework is therefore essential—not only to enhance clinical decision-making but also to facilitate a more comprehensive interpretation of outcomes, particularly in distinguishing recovery from compensation processes. Furthermore, rehabilitation research increasingly acknowledges the value of structured models for characterizing the mechanisms underlying motor recovery and compensatory strategies following stroke. Further clinical and preclinical research is needed to identify optimal time windows and strategies to improve true recovery rather than compensation (36).

In 2017, Kwakkel et al. affirmed the need of a consensus about a core set of valid measures to use in every stroke recovery and rehabilitation trial and the necessity to objectively measure quality of motor performance using technology to help distinguish restitution and compensation, established the “Stroke Recovery and Rehabilitation Round-table” (SRRR) to develop recommendations for standardized assessment times and measures to be included in all adult clinical trials of sensorimotor recovery after stroke. They organized three consensus meetings (23–25) on measurement standards and patient characteristics, clinical predictors, and pre-stroke clinical data to be collected in future stroke recovery trials. In these studies, recommendations are made based on time post-stroke and are aligned with the International Classification of Functioning, Disability, and Health. In the third roundtable (25), the group agreed on definitions related to balance and mobility and recommended a core set of outcome measures for lower-extremity motor function, balance and mobility, biomechanical metrics, and technologies for measuring quality of movement. Following this work, our study used a Delphi methodology, which has several features, such as the anonymity of the panelists and the statistical analysis of the results, focusing in depth on the two functions of gait and balance, distinguishing between recovery and compensation measures at the three ICF levels, including the neural level. We hope our study will contribute to the evidence base for precision stroke rehabilitation in these two functions.

As introduced by Hart et al. in 2019 (37, 38), the Rehabilitation Treatment Specification System (RTSS) is a structured framework for defining rehabilitation interventions based on their specific goals, active ingredients, and underlying mechanisms of action, whether aimed at promoting true recovery or facilitating compensatory strategies. This model emphasizes the formulation of rehabilitation strategies based on explicitly defined treatment goals, helping rehabilitators to select interventions based on an understanding of motor control that derives not only from functional outcome measures, but also from the integration of clinical data assessing body structure and function, as well as instrumented assessment of movement quality and neural alterations. The use of standardized assessment tools aligned with the RTSS framework, the identification of which is the objective of the present study, could support stratification of patients based on their functional and neurobiological profiles, thereby promoting precision rehabilitation approaches tailored to optimize recovery or, when appropriate, to compensate for deficits.

Establishing a shared conceptual and theoretical framework could facilitate the appropriate use of assessment and treatment tools aligned with different categories of the ICF. This enables investigation of their interrelations and effects across domains—particularly with neurophysiological biomarkers. In this context, the consensus statements from the International Stroke Recovery and Rehabilitation Alliance (ISRRA) (39) highlight the need to develop biomarkers of sensorimotor impairment for both clinical decision-making and research purposes. In addition to established biomarkers of stroke recovery, more precise biomarkers of sensorimotor impairment could enhance the prediction of rehabilitation outcomes, support more accurate goal setting, and inform the selection of individualized treatment strategies (39).

In a 2019 review, Balkaya M highlights the role of gait and kinematic analyses of locomotion and skilled reaching in providing sensitivity and enabling the detection of compensatory patterns to distinguish between recovery and compensation. While dedicated setups for such analysis exist, kinematic analysis can also be achieved using low-cost tools (36).

In addition to its clinical relevance, the present study also contributes methodologically to the field of rehabilitation research. Although the Delphi method has been widely used in healthcare for guideline development and consensus building, its application in rehabilitation, particularly in neurological conditions, remains relatively limited, with only a few studies adopting this approach (21, 40). The Delphi methodology employed in this study will serve as a basis for future studies, including a cross-sectional study to determine the prevalence of gait and balance disorders and to stratify stroke patients into clinically relevant subgroups, thereby promoting the development of precision rehabilitation strategies. Furthermore, the results will contribute to longitudinal investigations of gait and balance rehabilitation, enabling correlations between therapeutic interventions and changes in functional performance, movement quality, and neurobiological parameters.

This work will provide a cohort of tools that can be used according to the suggested methodology to distinguish between true recovery and compensation during gait and balance. The aim of this Delphi study is to develop a set of tools that will help all clinicians and researchers, including those in low-resource or non-specialist settings, select the most appropriate tool for their specific context, based on the evidence produced by this Delphi study.

### 3.1 Limits of the study

We acknowledge several limitations inherent to the present Delphi methodology. First, the consensus reached will reflect the opinions of the selected expert panel and may not be generalizable to the entire community of stroke rehabilitation professionals. Second, there is a risk of expert attrition across multiple rounds, which we will aim to mitigate through clear communication and incentives to reduce dropouts. Third, Delphi studies may be susceptible to the dominance of prevailing paradigms, whereby experts tend to favor established approaches over emerging or innovative tools; we have attempted to mitigate this by including panelists from diverse professional backgrounds and by allowing participants to suggest additional tools not included in the initial list. Finally, the outcomes of this protocol are consensus-based recommendations, and their empirical validation in clinical practice will require further research.

## 4 Conclusion

This study aims to establish consensus among experts on the most appropriate assessment tools for evaluating gait and balance in people with stroke, within a standardized framework that includes functional outcomes, movement quality, and neurobiological mechanisms, and that addresses both recovery and compensatory processes. This standardized approach aims to facilitate the consistent application of selected outcome measures in clinical and research settings. The consensus achieved through this Delphi process will provide a solid foundation for implementing precision rehabilitation in stroke, with the ultimate goal of improving rehabilitation outcomes and optimizing functional recovery.
